# Renal sympathetic nerve activity and vascular reactivity to phenylephrine after lipopolysaccharide administration in conscious rats

**DOI:** 10.14814/phy2.13139

**Published:** 2017-02-27

**Authors:** Claude Julien, Valérie Oréa, Luc Quintin, Vincent Piriou, Christian Barrès

**Affiliations:** ^1^ EA 7426: Pathophysiology of Injury‐Induced Immunosuppression (PI^3^)Faculty of PharmacyUniversity Claude Bernard Lyon 1LyonFrance; ^2^ Technical platform ANIPHYCNRS UMS 3453University Claude Bernard Lyon 1LyonFrance; ^3^ Department of PhysiologyUniversity Claude Bernard Lyon 1LyonFrance; ^4^ Anesthesiology/Critical CareHôpital d'Instruction des Armées DesgenettesLyonFrance; ^5^ Department of Anesthesiology and Intensive CareLyon‐Sud HospitalHospices Civils de LyonLyonFrance; ^6^ University Claude Bernard Lyon 1LyonFrance

**Keywords:** Dexmedetomidine, endotoxemia, LPS, sepsis, *α*_2_‐adrenoceptor agonist

## Abstract

It has been proposed that sympathoexcitation is responsible for vascular desensitization to *α*_1_‐adrenoceptor stimulation during lipopolysaccharide (LPS)‐induced systemic inflammation. The present study tested this hypothesis by examining the effects of sympatho‐deactivation with the *α*_2_‐adrenoceptor agonist, dexmedetomidine, on mean arterial pressure (MAP), renal sympathetic nerve activity (RSNA), and vascular reactivity to phenylephrine in conscious rats with cardiac autonomic blockade (methylatropine and atenolol) following LPS administration. In male, adult Sprague‐Dawley rats (*n *=* *5 per group), RSNA and MAP were continuously recorded over 1‐h periods, before and after LPS administration (20 mg/kg iv), and finally after infusion of either saline or dexmedetomidine (5 *μ*g/kg, then 5 *μ*g/kg/h iv). A full dose**–**response curve to phenylephrine was constructed under each condition. After pooling data from both groups of rats (*n *=* *10), LPS significantly (*P *=* *0.005) decreased MAP (from 115 ± 1 to 107 ± 2 mmHg), increased RSNA (to 403 ± 46% of baseline values) and induced 4 to 5‐fold increases in the half‐maximal effective dose (ED_50_) of phenylephrine (from 1.02 ± 0.09 to 4.76 ± 0.51 *μ*g/kg). During saline infusion, RSNA progressively decreased while vascular reactivity did not improve. Treatment with dexmedetomidine decreased MAP, returned RSNA to near pre‐endotoxemic levels, but only partially restored vascular reactivity to phenylephrine (ED_50_ was still threefold increased as compared with baseline values). These findings indicate that only part of the decrease in vascular reactivity to *α*_1_‐adrenoceptor stimulation during endotoxemia can be accounted for by sympathetic activation, at least on a short‐term basis.

## Introduction

Systemic inflammation is a major component of endotoxemic shock and is accompanied by decreased arterial pressure (AP) responsiveness to vasopressors, especially noradrenaline (Andreis and Singer 2016). It has been hypothesized that massive sympathetic activation during endotoxemic shock might lead to desensitization of vascular *α*_1_‐adrenoceptors, and that sympathetic inhibition with centrally acting sympatholytic drugs such as clonidine and dexmedetomidine might be effective in restoring vascular reactivity and hence, might improve AP control (Pichot et al. 2010; Geloën et al. 2015). Geloën and coworkers reported that rats challenged with lipopolysaccharide (LPS) had decreased pressor responses to noradrenaline that could be entirely restored by clonidine and dexmedetomidine (Geloën et al. 2013). This important study, however, suffered from several limitations (Dünser and Gradwohl‐Matis 2013; Geloën et al. 2013). First, sympathetic nerve activity (SNA) was not assessed. Second, experiments were performed in rats anesthetized with isoflurane, which probably altered autonomic cardiovascular control (Shimokawa et al. 1998; Akata et al. 2003). Third, doses of clonidine and dexmedetomidine (200 and 100 *μ*g/kg iv, respectively) that were administered to rats were extremely large by comparison with those presently used for sedation of endotoxemic patients (range: 0.5–1.5 *μ*g/kg/h) (Pichot et al. 2012). Fourth, the chronotropic and inotropic control of the heart by autonomic nerves was not interrupted, so that changes in pressor responsiveness could not be safely attributed to changes in vascular responses only. Fifth, full dose**–**response curves were not analyzed because doses of noradrenaline required to reach the plateau of the response were almost invariably lethal in this model (Geloën et al. 2013).

The present study was designed to circumvent these limitations. Renal SNA (RSNA) was measured in conscious, chronically instrumented rats. Dexmedetomidine was administered at a lower dose (5 *μ*g/kg, then 5 *μ*g/kg/h), which has been shown to markedly improve survival 8 h after endotoxin injection in pentobarbital‐anesthetized rats (Taniguchi et al. 2004). All experiments were carried out under cardiac autonomic blockade with methylatropine and atenolol, so that changes in pressor responses to phenylephrine could be attributed only to changes in vascular responses to *α*_1_‐adrenoceptor stimulation. Atenolol is a selective *β*_1_‐adrenoceptor antagonist and thus, is supposed not to interfere with the inflammatory response to endotoxin which is mainly mediated by *β*_2_‐adrenoceptors (Lorton and Bellinger 2015). Finally, full dose**–**response curves to phenylephrine were constructed, allowing determination of the plateau of the response together with its sensitivity.

## Materials and Methods

### Animals and surgery

All experiments were performed on the technical platform ANIPHY after approval by the Ethics Committee for Animal Experimentation of University Claude Bernard Lyon 1. Male Sprague‐Dawley rats (300–350 g; Charles River Laboratories, L'Arbresle, France) were housed individually at least 3 days before commencing experiments for acclimatization. They were maintained under controlled conditions (temperature 22°C; lights on 07:00–19:00 h) with ad libitum access to standard food pellets and tap water. One day before study, under isoflurane anesthesia (2% in oxygen), one catheter was inserted into the abdominal aorta through a femoral artery for AP measurement, and two catheters were inserted into the inferior vena cava through the ipsilateral femoral vein for drug administration. Catheters were routed subcutaneously and exteriorized between the scapulae. To avoid ischemia of the leg, rats were allowed to recover from anesthesia and to regain full motility. Four to 6 h after catheterization, rats were reanesthetized with pentobarbital sodium (60 mg/kg iv) for implantation of the renal nerve electrode (Bertram et al. 2005). To alleviate post‐operative pain, the nonsteroidal anti‐inflammatory drug carprofen (5 mg/kg sc) and buprenorphine (0.05 mg/kg sc) were administered before and after surgery respectively. Through a flank incision, a branch of the left renal sympathetic nerve was dissected, placed on a bipolar platinum‐iridium electrode and embedded in silicone gel. The electrode cable was secured to back muscles and exteriorized at the same site as the catheters. The connector plug was protected in a small cap sewn to the skin.

### Experimental protocol

**Figure 1:**
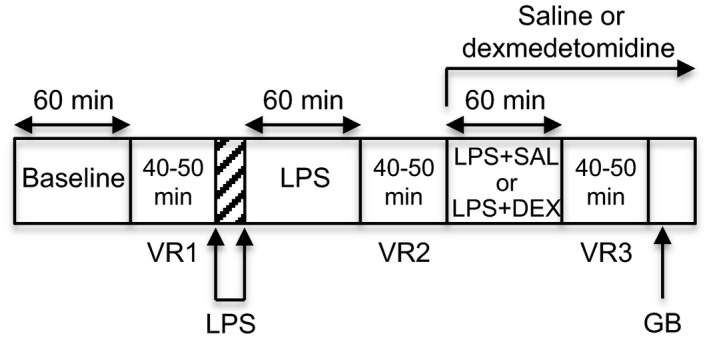
Time course of the experiment. After a 60‐min baseline period, vascular reactivity (VR) was assessed by generating a pressor dose**–**response curve to phenylephrine. Lipopolysaccharide (LPS, 20 mg/kg iv) was then administered over 15 min (hatched area), followed by a 60‐min observation period. After a second vascular reactivity assessment, either saline (SAL) or dexmedetomidine (DEX, 5 *μ*g/kg, then 5 *μ*g/kg/h) was infused until the end of the experiment (*n *=* *5 in each group: LPS + SAL or LPS + DEX). After another 60‐min observation period and a third vascular reactivity assessment, ganglionic blockade (GB) was achieved with chlorisondamine (2.5 mg/kg iv) before euthanasia with pentobarbital.

Sixteen to 18 h after completion of the nerve electrode implantation, the arterial catheter and the nerve electrode were connected to the recording system (Bertram et al. 2005). The renal sympathetic nerve activity (RSNA) signal was amplified (×50,000) and bandpass filtered at 300–3,000 Hz. This raw signal was directly sampled at 10,000 Hz. It was also full‐wave rectified and low‐pass filtered at 150 Hz by an analog custom‐made rectifier before being sampled at 5000 Hz (Chapuis et al. 2010). The AP signal was sampled at 500 Hz. Data acquisition was performed using LabVIEW 5.1 software (National Instruments, Austin, TX). Throughout the experiment, cardiac autonomic blockade was achieved with the combined administration of methylatropine and atenolol (2 mg/kg each followed by 2 mg/kg/h iv). AP and RSNA were first recorded during 1 h (referred to as baseline period, Fig. 1). Then, a pressor dose**–**response curve to phenylephrine was established to assess vascular reactivity. Increasing bolus doses of phenylephrine (10–12 doses ranging from 0.19 to 12 *μ*g/kg iv) were administered. The highest dose of phenylephrine was chosen to evoke an increase in AP close to that evoked by the previous, lower dose so as to ensure that the plateau of the response was reached. Between consecutive doses, sufficient time (5–10 min) was allowed for AP to return to its pre‐injection level. Endotoxemia was then induced by iv infusion of LPS (20 mg/kg over 15 min, Fig. 1). After completion of the infusion, a 60‐min recording was performed, after which a second dose**–**response curve to phenylephrine was generated (10–14 doses ranging from 0.38 to 36 *μ*g/kg iv). Afterward, saline (0.12 mL, then 1 mL/kg/h iv) or dexmedetomidine (5 *μ*g/kg, then 5 *μ*g/kg/h iv) was infused until the end of the study. After a further 60‐min recording, a third dose**–**response curve to phenylephrine was established (12–14 doses ranging from 0.19 to 24 *μ*g/kg iv). At the end of the recording session, the ganglionic blocker chlorisondamine was administered (2.5 mg/kg iv) to determine the background noise in the RSNA signal. In addition, the change in mean AP (MAP) induced by chlorisondamine was used to quantify the functional importance of SNA in sustaining MAP on a short‐term basis. Finally, rats were euthanized with pentobarbital sodium (>100 mg/kg iv). LPS (from *E. coli* 0111:B4) was obtained from Sigma‐Aldrich (Saint‐Quentin‐Fallavier, France), and dexmedetomidine (Dexdomitor^®^) from Orion Janssen (Issy‐les‐Moulineaux, France).

### Data analysis

AP and rectified RSNA time series were resampled at 1 Hz by averaging data over consecutive 1‐s periods. One‐s average values of AP are referred to as MAP values in the following of the text. Within each 1‐s segment, heart rate (HR) was calculated from the mean interbeat interval. The background noise level of RSNA was taken as the mean RSNA value calculated over a 2‐min period following chlorisondamine. This value was subtracted from all RSNA data for subsequent analyses. Under each condition (Fig. 1), i.e., baseline (before LPS administration), LPS (1 h after LPS administration), LPS + SAL (after LPS administration plus 1 h after starting saline infusion), LPS + DEX (after LPS administration plus 1 h after starting dexmedetomidine infusion), representative MAP, HR and RSNA values were estimated as the 10‐min average values preceding a dose**–**response curve to phenylephrine. Peak increases in MAP induced by phenylephrine were calculated as the maximum changes of MAP from its value recorded immediately before injection. These pressor responses were plotted against the log_10_ of the corresponding phenylephrine dose. Because the MAP phenylephrine dose**–**response relationship frequently showed asymmetry of curvature, we systematically fitted a five‐parameter logistic equation to the data (SigmaPlot 2000; Systat Software, San Jose, CA). The equation actually reduces to a four‐parameter equation because it is forced to zero (i.e., there is no MAP change when the phenylephrine dose is null). The equation is therefore

$$y=\frac{a}{{\left(1+e^{-\frac{x-x_{0}}{b}}\right)}^{c}}$$

where y is the MAP change, x is the log_10_ of the phenylephrine dose, a is the plateau of the MAP response (maximum effect), b and c are curvature coefficients, and

$$x_{0}={\text{ED}}_{50}+\text{bLn}\left(2^{\frac{1}{c}}-1\right)$$

where ED_50_ is the log_10_ of the half‐maximal effective dose and provides the sensitivity of the response after inverse log transformation (it is then expressed in *μ*g/kg).

### Statistics

All data are reported as mean ± SE. Within each group of rats, comparisons between conditions were performed using the Friedman test for repeated measures followed by a Wilcoxon signed‐rank test for pairwise comparisons, each time the Friedman test was significant. The Mann‐Whitney test was used for unpaired between‐group comparisons. Regarding RSNA, paired comparisons were performed using absolute values (in *μ*V), and unpaired comparisons were performed using percentage of baseline values.

## Results

### Effect of LPS on cardiovascular variables and vascular reactivity over one hour

**Figure 2:**
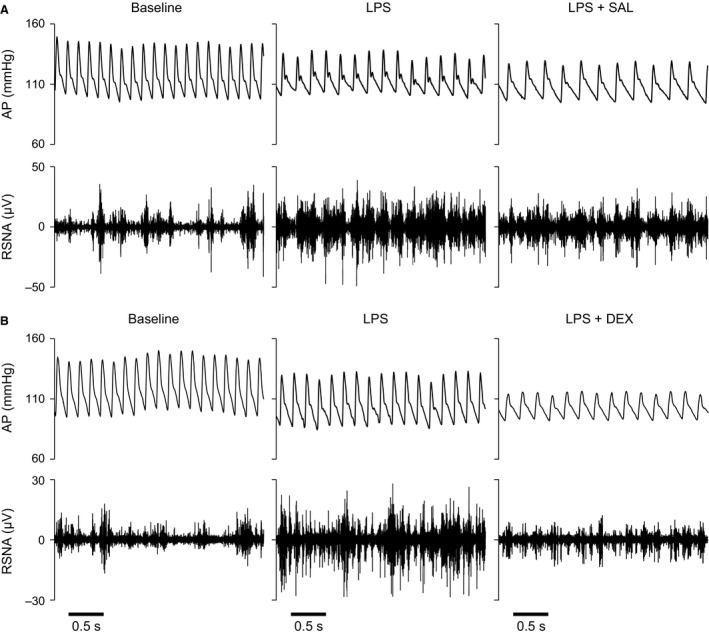
Examples of 3‐s recordings of arterial pressure (AP) and renal sympathetic nerve activity (RSNA, raw signal before analog rectification) in two conscious rats before (Baseline), 1 h after induction of endotoxemia with lipopolysaccharide (LPS), and after induction of endotoxemia then subsequent infusion of saline (LPS + SAL) (A), or dexmedetomidine (LPS + DEX) (B).

Shortly after starting the LPS infusion, MAP dropped, sometimes by 40–50 mmHg while RSNA increased abruptly. Then, MAP gradually returned toward baseline values (within 10–15 min after the end of the infusion) while RSNA remained elevated. Finally, both MAP and RSNA tended to decline. During the baseline period and after induction of endotoxemia, MAP, HR and RSNA did not differ between the two groups of rats (Table 1). After pooling these data, it was observed that LPS decreased MAP (from 115 ± 1 to 107 ± 2 mmHg, *n *=* *10, *P *=* *0.005) and HR (from 375 ± 7 to 346 ± 9 bpm, *n *=* *10, *P *=* *0.013), and increased RSNA (to 403 ± 46% of baseline values, *n *=* *10, *P *=* *0.005) (Fig. 2).

**Table 1:** Mean values of cardiovascular variables under various experimental conditions

	Baseline	LPS	LPS + SAL/DEX	*P*
MAP (mmHg)				
SAL Group (*n *=* *5)	115 ± 2	110 ± 2*	95 ± 6*	0.015
DEX Group (*n *=* *5)	116 ± 2	105 ± 3*	86 ± 7*, †	0.007
* P*	0.917	0.251	0.251	
HR (bpm)				
SAL Group (*n *=* *5)	371 ± 11	360 ± 9	378 ± 4	0.247
DEX Group (*n *=* *5)	378 ± 10	331 ± 15*	306 ± 17*, †	0.007
* P*	0.753	0.117	0.009	
RSNA (% baseline)				
SAL Group (*n *=* *5)	100	462 ± 75*	255 ± 63*, †	0.007
DEX Group (*n *=* *5)	100	345 ± 54*	135 ± 19†	0.015
* P*	—	0.251	0.047	

Values are means ± SE. LPS, lipopolysaccharide; SAL, saline; DEX, dexmedetomidine; MAP, mean arterial pressure; HR, heart rate; RSNA, renal sympathetic nerve activity. *P* values contained in the column refer to paired comparisons (Friedman test). *P* values contained in the lines refer to unpaired comparisons (DEX group vs. SAL group, Mann‐Whitney test). *P <* *0.05 vs. baseline values and ^†^ *P *<* *0.05 vs. values after LPS administration (Wilcoxon signed‐rank test).

**Figure 3:**
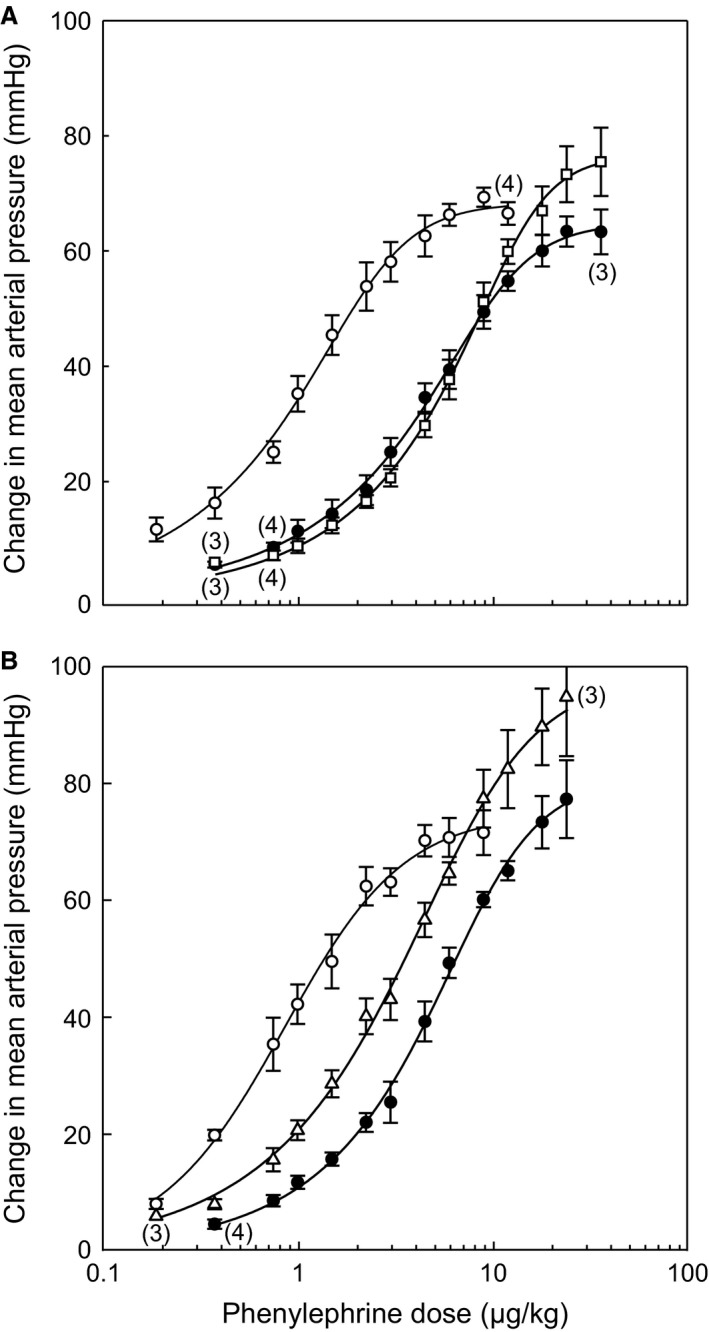
Effect of treatment with saline (*n *=* *5, A) or dexmedetomidine (*n *=* *5, B) on dose**–**response curves to phenylephrine in conscious rats before (open circles), 1 h after induction of endotoxemia with lipopolysaccharide (closed circles), and 1 h after starting infusion of saline (open squares) or dexmedetomidine (open triangles). Each point is the mean ± SE of 5 determinations, unless indicated in parentheses. For illustrative purpose, curves were fitted to these data points using a 5‐parameter logistic equation.

Dose**–**responses curves to phenylephrine could be satisfactorily fitted under all conditions (Fig. 3; coefficients of determination (r^2^) range: 0.977 ± 0.011–0.996 ± 0.001). As neither the maximum response nor the sensitivity of the response differed between groups during baseline period and after LPS administration (Table 2), values were pooled. LPS did not change the maximum response to phenylephrine (from 73.3 ± 2.6 to 72.6 ± 3.8 mmHg, *n *=* *10, *P *=* *0.879) but strongly increased the ED_50_ (from 1.02 ± 0.09 to 4.76 ± 0.51 *μ*g/kg, *n *=* *10, *P *=* *0.005).

**Table 2:** Main characteristics of dose**–**response curves to phenylephrine under various experimental conditions

	Baseline	LPS	LPS + SAL/DEX	*P*
Plateau (mmHg)				
SAL Group (*n *=* *5)	70.4 ± 2.7	68.2 ± 3.8	76.3 ± 6.3	0.549
DEX Group (*n *=* *5)	76.2 ± 4.5	77.1 ± 6.3	94.5 ± 5.5*, †	0.022
*P*	0.251	0.347	0.117	
ED_50_ (*μ*g/kg)				
SAL Group (*n *=* *5)	1.12 ± 0.12	4.74 ± 0.68*	6.20 ± 0.91*	0.015
DEX Group (*n *=* *5)	0.91 ± 0.14	4.78 ± 0.84*	3.20 ± 0.61*, †	0.007
*P*	0.117	0.917	0.028	

Values are means ± SE. LPS, lipopolysaccharide; SAL, saline; DEX, dexmedetomidine; ED_50,_ half‐maximal effective dose. *P* values contained in the column refer to paired comparisons (Friedman test). *P* values contained in the lines refer to unpaired comparisons (DEX group vs SAL group, Mann**–**Whitney test). *P <* *0.05 vs. baseline values and ^†^ *P *<* *0.05 vs. values after LPS administration (Wilcoxon signed‐rank test).

### Effect of saline and dexmedetomidine on cardiovascular variables and vascular reactivity in endotoxemic rats

During saline infusion, MAP tended to decrease further (*P *=* *0.08), whereas HR did not vary significantly. In the meantime, RSNA showed a clear decrease. At the end of the observation period (50–60 min after starting saline infusion), RSNA was almost halved but remained significantly elevated over baseline values (Table 1 and Fig. 2). The bolus injection of dexmedetomidine (5 *μ*g/kg iv) induced a transient, short‐lasting (<10 min) increase in MAP. Afterward, MAP and HR progressively declined and reached significantly lower values than baseline levels at the end of the observation period, while RSNA returned to values that did not significantly differ from baseline values (Table 1 and Fig. 2).

During saline infusion, the maximum response to phenylephrine and the ED_50_ did not vary significantly (Table 2). During dexmedetomidine infusion, the plateau of the response was significantly elevated over both baseline values and values observed after LPS before starting dexmedetomidine infusion (Table 2), so that MAP values reached after administration of the highest doses of phenylephrine (180 ± 5 mmHg) did not significantly differ (*P *=* *0.117) from those reached during saline infusion (172 ± 2 mmHg). By contrast, the ED_50_ was significantly lowered compared to values measured after LPS before dexmedetomidine administration, but remained significantly increased over baseline values (Table 2 and Fig. 3).

### Effect of dexmedetomidine on mean arterial pressure response to chlorisondamine in endotoxemic rats

In the group of rats infused with saline (*n *=* *5), administration of chlorisondamine at the end of the study decreased MAP by 38.0 ± 7.2 mmHg. In the group of rats receiving the dexmedetomidine infusion (*n *=* *5), chlorisondamine induced a significantly lower drop in MAP (14.6 ± 3.4 mmHg, *P *=* *0.047).

## Discussion

In conscious rats, acute LPS administration induces renal sympathoexcitation and decreases vascular reactivity to *α*_1_‐adrenoceptor stimulation with phenylephrine. Dexmedetomidine treatment reverses sympathoexcitation but only partly restores vascular reactivity to phenylephrine, thus suggesting that overstimulation of *α*_1_‐adrenoceptors by endogenously released noradrenaline is only partially responsible for their desensitization.

### Cardiovascular effects of lipopolysaccharide in conscious rats

There has been one previous report on the effect of endotoxemia induced by *E. coli* endotoxin (20 mg/kg iv) on the RSNA of conscious rats (Pålsson et al. 1988). The kinetics and amplitude of the RSNA increase were comparable to those observed in our experiments (an immediate 200% increase lasting at least 60 min). The mechanisms of endotoxemia‐induced sympathoexcitation are still unclear. Classically, an important role of vagal afferents has been assumed (Andersson and Tracey 2012). Recently, however, Martelli et al. (2014) reported that bilateral cervical vagotomy did not affect the rise in splanchnic SNA observed in urethane‐anesthetized rats after injection of LPS (60 *μ*g/kg iv), thus favoring a direct role of humoral influences on central nervous structures controlling SNA (Kannan et al. 1996; MacNeil et al. 1997). As to the role of the baroreceptor reflex, it has been ruled out by at least two studies. In the first one performed on conscious rats (Pålsson et al. 1988), LPS induced an initial transient decrease in MAP which then returned to normal while the increase in RSNA remained unchanged. Even more direct evidence was gained in a study carried out on pentobarbital‐anesthetized rats (Vayssettes‐Courchay et al. 2005), where it was demonstrated that arterial baroreceptor denervation minimally affected the RSNA increase induced by LPS (20 mg/kg iv).

After recovery from the initial drop induced by LPS, we observed a progressive, albeit moderate, decrease in MAP. This effect was observed all along the experiment but was especially marked during saline infusion. In the study by Pålsson and colleagues (Pålsson et al. 1988), MAP was well‐maintained until the end of the experiment (90 min after endotoxemia induction). It is of note, however, that in the latter study, rats did not receive cardiac autonomic blockers so that HR was free to increase by about 100 bpm, which could have helped maintain MAP. In the present study, HR decreased after LPS administration. This probably resulted from the direct action of LPS on the sinus node (Ebelt et al. 2015) and/or from the occurrence of hypothermia (Romanovsky et al. 2005), which is known to decrease intrinsic HR (Bolter and Atkinson 1988). Because all rats were euthanized at the end of the recordings, we do not know whether MAP would have continued to decrease. In two previous studies on pentobarbital‐anesthetized rats, MAP was reported to decrease continuously after LPS administration. Rats died either between 3 and 5 h post endotoxemia induction when LPS was given at the dose of 20 mg/kg iv (Vayssettes‐Courchay et al. 2005) or between 3 and 8 h when LPS was given at the dose of 15 mg/kg iv (Taniguchi et al. 2004). By contrast, in the study by Geloën and colleagues (2013) on isoflurane‐anesthetized rats, AP was not lowered 1 h after LPS infusion (30 mg/kg iv). This is consistent with a previous study reporting that isoflurane blunts the inflammatory response to LPS administration and increases the survival time in mice (Fuentes et al. 2006).

Whatever the mechanism of the progressive hypotension in the endotoxemic rats of the present study, it is interesting to note that MAP was sustained by SNA until the end of the observation period. This is suggested by the parallel decreases in MAP and RSNA occurring during saline infusion. Furthermore, the magnitude of the depressor effect of acute ganglionic blockade that was measured at the very end of the experiment was only slightly less than that previously reported in conscious, male Sprague‐Dawley rats (Santajuliana et al. 1996).

### Effects of lipopolysaccharide on vascular reactivity to phenylephrine in conscious rats

One hour after endotoxemia induction, there was a 4 to 5‐fold increase in the ED_50_ of the pressor response to phenylephrine. This vascular hyporeactivity to phenylephrine in conscious rats is of the same order of magnitude than the decrease in pressor responsiveness to noradrenaline previously reported by Geloën and colleagues in anesthetized rats (2013). The maximum pressor response to phenylephrine was unaltered in the endotoxemic rats of the present study, which would be compatible with an agonist‐mediated desensitization of vascular *α*_1_‐adrenoceptors. One intriguing observation, however, is that after RSNA had decreased by almost 50% during saline infusion, dose**–**response curves to phenylephrine remained shifted to the right, i.e., no resensitizitation had occurred. This suggests that the average level of SNA might not be a strong determinant of adrenergic vascular reactivity under these conditions.

### Effects of dexmedetomidine in conscious, endotoxemic rats

In pilot experiments, we sought to determine a dose of dexmedetomidine that would restore RSNA to or near normal levels in conscious, endotoxemic rats. In our first attempts, we administered dexmedetomidine at the dose of 25 *μ*g/kg/h. This dose completely abolished RSNA. With the dose of 5 *μ*g/kg as a bolus injection followed by the continuous infusion of 5 *μ*g/kg/h, there was first a transient pressor effect, probably resulting from the acute stimulation of vascular, extrasynaptic *α*_2_‐adrenoceptors (Timmermans and Van Zwieten 1980; Talke et al. 2003). Then, MAP and RSNA decreased in parallel. By the end of the observation period (50–60 min after starting dexmedetomidine treatment), RSNA had returned toward baseline levels and MAP had decreased by 20 mmHg. After completion of the last testing of vascular reactivity to phenylephrine, RSNA and MAP had slightly further decreased (data not shown). The depressor effect of ganglionic blockade was strongly attenuated but remained measurable, which demonstrated the dependency of MAP on SNA. Finally, it must be noted that dexmedetomidine decreased HR, which also possibly contributed to hypotension in endotoxemic rats. This effect on HR might be due to the inhibitory effect of dexmedetomidine on the discharge of pacemaker cells in the sinus node, which has been demonstrated in isolated rabbit hearts (Pan et al. 2015).

One of the most important findings of this study is that the vascular reactivity to *α*_1_‐adrenoceptor stimulation with phenylephrine after dexmedetomidine administration was improved, but far from fully restored (the ED_50_ remained increased by a factor of 3 as compared with baseline). This observation is clearly at variance with that by Geloën and colleagues (2013), who reported a complete restoration of pressor responses to noradrenaline after dexmedetomidine administration (100 *μ*g/kg iv). As mentioned above, even a much lower dose (25 *μ*g/kg iv) was able to fully abolish RSNA. It is therefore conceivable that complete sympatholysis might have much more marked effects on vascular reactivity to *α*_1_‐adrenoceptor agonists. One puzzling observation, however, is that Geloën and coworkers reported that dexmedetomidine did not decrease AP in their endotoxemic rats (Geloën et al. 2013), which suggests that there was little or no sympathetic tone sustaining AP before the administration of the *α*_2_‐adrenoceptor agonist. This makes it difficult to compare the results of the two studies. It remains that the present study indicates that short‐term exposure to sympathetic hyperactivity can only partly explain the decreased vasoconstrictor responses to an *α*_1_‐adrenoceptor agonist in conscious, endotoxemic rats. From a clinical standpoint, the question arises as to whether a longer treatment time with dexmedetomidine would have led to a further improvement in vascular reactivity to phenylephrine. While the question deserves to be examined, that would imply that the kinetics of desensitization and resensitization markedly differ from each other, which has not been reported in the literature.

An interesting finding is that the plateau of the response was increased after dexmedetomidine administration so that the maximum MAP values reached after phenylephrine injections were similar to those measured in saline‐infused rats. This indicates that after dexmedetomidine, it is possible to raise AP with very large doses of phenylephrine in endotoxemic rats. However, caution is warranted, as this conclusion applies to bolus injections of phenylephrine and by no means should be extended to continuous infusions of phenylephrine.

A last point that deserves discussion is the finding by Lankadeva et al. (2015) that clonidine restores pressor responses to both phenylephrine and angiotensin II in a model of chronic sepsis in the conscious sheep. Although it cannot be excluded that this action is specific to clonidine, it should be considered as a possible effect of dexmedetomidine when designing future experiments. While this suggests a non‐selective action of *α*_2_‐adrenoceptor agonists on vascular reactivity (Kawahito et al. 2011; Kawano et al. 2012), an alternative, but not exclusive, explanation could be that clonidine, through its inhibitory action on RSNA, decreases the release of renin and the ensuing formation of angiotensin II, which have been shown to be strongly increased during sepsis (Schaller et al. 1985; Doerschug et al. 2010). As a consequence, vasoconstrictor responses to angiotensin II would be enhanced. Unfortunately, in this study, plasma renin activity or angiotensin II concentrations were not measured (Lankadeva et al. 2015).

## Conclusions

The present study does not bring strong support to the hypothesis that sympathetic hyperactivity is responsible for desensitization of vascular *α*_1_‐adrenoceptors during endotoxemia (Pichot et al. 2012; Geloën et al. 2015). Nevertheless, dexmedetomidine improved vascular reactivity to phenylephrine, through a mechanism that largely remains to be determined. However, this effect was achieved at the cost of a decrease in MAP, suggesting that enhanced vasoconstrictor responses to *α*_1_‐adrenoceptor stimulation were unable to fully compensate for the decrease in SNA induced by dexmedetomidine. The diminished depressor effect of ganglionic blockade also speaks in favor of this interpretation.

## Conflict of Interest

L. Quintin holds a US patent 8 846 606 B2, Sept 30, 2014, on method and drug composition for treating endotoxemic shock hypotension.
